# Bacterial Meningitis: A Density-Equalizing Mapping Analysis of the Global Research Architecture

**DOI:** 10.3390/ijerph111010202

**Published:** 2014-09-30

**Authors:** Niklas Pleger, Beatrix Kloft, David Quarcoo, Simona Zitnik, Stefanie Mache, Doris Klingelhoefer, David A Groneberg

**Affiliations:** 1 Institute of Occupational Medicine, Charité—School of Medicine, Humboldt University Berlin, Thielallee 73, 14195 Berlin, Germany; E-Mail: niklas.pleger@charite.de; 2 Unit of Health Economics, Department of Gynecology and Obstetrics, Goethe University Frankfurt, Theodor-Stern-Kai 7, 60590 Frankfurt, Germany; E-Mail: beakloft@gmx.de; 3 Institute of Occupational Medicine, Social Medicine and Environmental Medicine, Goethe University Frankfurt, Theodor-Stern-Kai 7, 60590 Frankfurt, Germany; E-Mails: quarcoo@web.de (D.Q.); arbsozmed@uni-frankfurt.de (S.Z.); mache@med.uni-frankfurt.de (S.M.); klingelhoefer@med.uni-frankfurt.de (D.K)

**Keywords:** bacterial meningitis, acute meningitis, scientometric analysis, density equalizing mapping

## Abstract

Bacterial meningitis is caused by a variety of pathogens and displays an important public health threat all over the world. Despite the necessity to develop customized public health-related research projects, a thorough study of global meningitis research is not present, so far. Therefore, the aim of this study was a combined density-equalizing and scientometric study. To evaluate the scientific efforts of bibliometric methods, density-equalizing algorithms and large-scale data analysis of the Web of Science were applied in the period between 1900 and 2007. From this, 7998 publications on bacterial meningitis have been found. With a number of 2698, most publications have been written by U.S. authors, followed by the UK (912), Germany (749) and France (620). This dominance can also be shown in the international cooperation. The specific citation analyses reveal that the nation with the highest average citation rate (citations per publications) was Norway (26.36), followed by Finland (24.16) and the U.S. (24.06). This study illustrates the architecture of global research on bacterial meningitis and points to the need for customized research programs with a focus on local public health issues in countries with a low development index, but high incidences, to target this global public health problem.

## 1. Introduction

Meningitis is a severe medical condition. It is an important public health issue whether cases arises in a public health setting or sporadically in the community,* i.e.*, in schools or nursery schools [1,2]. From a global point of view, all issues related to its epidemiology, rapid diagnosis, economic treatment and prevention [3,4,5] are major public health issues, since meningitis affects children, adolescents and adults in all parts of the world [6,7].

Meningitis is defined as an inflammation of the protective membranes that surround the brain and the spinal cord. Hence, it involves the pia mater, the arachnoid and the cerebrospinal fluid (CSF) [8]; arachnoiditis or leptomeningitis are therefore also used to describe the sickness. The inflammation can have a bacterial, a viral, as well as an aseptic cause. Bacterial meningitis is a medical emergency associated with high rates of morbidity, mortality [9,10] and a wide range of different causative organisms. Its etiology is influenced by numerous factors, most of all by the age of the patient [11], but immunization status, immune function and geographic area of the infection are additionally relevant factors [12]. *Streptococcus pneumoniae*, *Haemophilus influenzae* type b (Hib) and *Neisseria meningitidis* cause the majority of cases of acute bacterial meningitis (ABM) in countries with a high development index [13,14], but the epidemiology of the disease is rapidly changing due to immunization practices and changing bacterial resistance patterns [12,15].

Next to an optimal therapy that needs to be directed against the causative agent and the symptoms [10,12], prevention has a high priority in the core of public health efforts [16].

Because of the global burden of meningitis, the need for geographically focused research programs with the emphasis on public health issues and the involvement of various disciplines of medicine, such as hygiene, pediatrics, neurology or infectiology, has a high priority.

So far, there has been no concise study that delineates the global meningitis research architecture. Therefore, the NewQIS platform [17,18] elected bacterial meningitis as a research focus with the aim of assessing and analyzing global research patterns.

## 2. Experimental Section 

### 2.1. Data Sources

In this study, a bibliometric analysis was performed to generate findings on scientometric parameters and indices. Data were retrieved from the Thomson Reuters Web of Science database (WoS), which is available online.

### 2.2. Search Strategies

The methods developed in the NewQIS project were used to retrieve and analyze the thematic entries from WoS by searching with the term “bacteria* meningiti*”. This procedure was refined by including only the Science Citation Index Expanded (SCIE) and the Social Sciences Citation Index (SSCI) in the search; whereas, the entries of the Arts and Humanities Citation Index (A&HCI) were excluded. As the time span, the period between 1900 until 2007 was chosen in order to get a closed and manageable period of time that does not distort the overall findings by the decline of the number of citations in the last few years due to the cited half-life of publications. In this respect, the main objective was to allow the publications included to have sufficient time in public in order to generate the maximum of attention in the scientific community, valuated by the summarized received citations.

### 2.3. Processing of Data

The results were gathered using the “output records” function of WoS [19,20]. Afterwards, the data were stored in our own database that made it possible to analyze the findings concerning a variety of different aspects, e.g., the publication date, the source of the title, the country of origin, the involved authors and the institutions, as well as the most productive journals.

### 2.4. Analysis of Citations

Specific citation analysis was performed as described,* i.e.*, by Gerber* et al.* [21]. In brief, to refine analysis, all items found with the search string were analyzed using the “citation report” function of WoS. This method was used to summarize the citations per year of all items, the citation rates of all given years and all involved countries, as well as the modified h-indices of countries and authors that are only related to the research area of bacterial meningitis and displays, not their overall publication performance.

### 2.5. Analysis of Cooperation

Additionally, the retrieved results were analyzed and categorized into various tables [22]. To perform an analysis of the cooperation network, the data were examined regarding the origin of the authors. The found entries were adjusted to an up-to-date list of countries, e.g., countries that are part of a state were combined. In this way, countries, such as Wales, Scotland, England and Northern Ireland, were summarized as UK, but in contrast, countries that currently no longer form a single state, like the Czech Republic and Slovakia, were separated. To illustrate the international network, an application was used that displays the countries and connects them with lines, whereby the thickness and color correspond to the number of cooperative efforts between the countries.

### 2.6. Density-Equalizing Mapping

To illustrate the results regarding geographical issues, e.g., the modified h-index (Hirsch-factor) or the productivity of institutes, the technique of density-equalizing mapping (DEMP) was used. These contorted global maps were generated using specific calculations based on the algorithm of Gastner and Newman. This method follows a principle of calculation, by means of which the value of a certain parameter, e.g., the average citation rate, was assigned to the various countries. The country size was then calculated, resulting in a re-sized map of the territory according to the geographical distribution of the said variable [23]. By this means, the results can be clearly and precisely shown as an anamorphic map.

### 2.7. H-Index

A modified, country-specific, h-index (Hirsch-factor) was applied to compare the scientific impact of countries among each other. A specific country is entitled to an h-index of *h*, if *h* of its *n* publications received at least *h* citations and the other papers (*n − h*) got less than *h* citations [24,25,26].

## 3. Results and Discussion

### 3.1. Results

#### 3.1.1. Analysis of the Global Research Output 

By using the defined search string, the analysis reveals 7998 publications on bacterial meningitis in the period examined.

**Figure 1:**
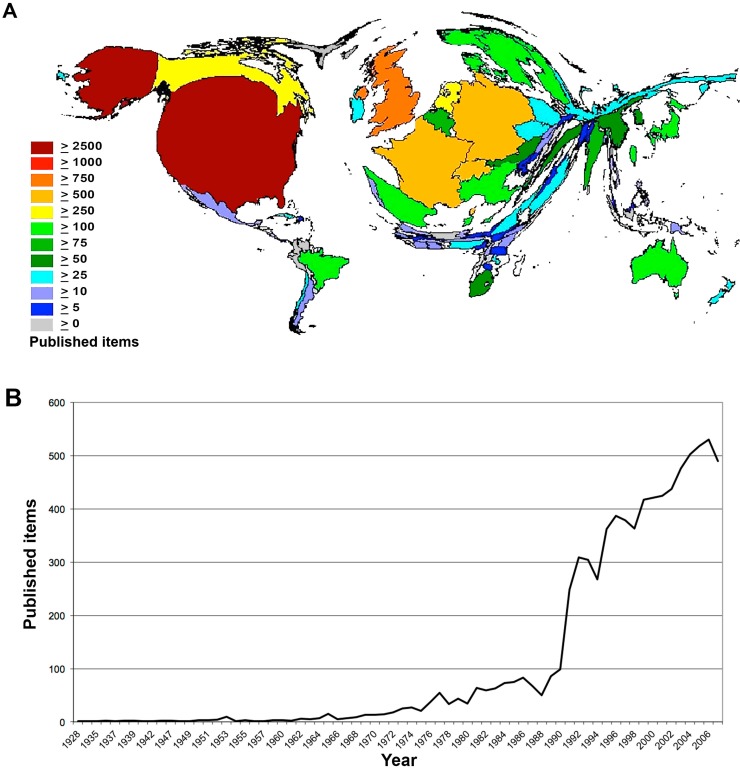
Global research activity. (**A**) Density-equalizing map of the numbers of published items. (**B**) Development of productivity over a time period of 79 years. In 1990, abstracts were included to the Web of Science (WoS).

The most productive country was the U.S., with 2698 articles on bacterial meningitis. A density-equalizing cartogram (Figure 1A) illustrates its dominance. The following most productive states were some countries of Europe. While the UK published 912 items, German authors wrote 749 publications and French authors contributed 620 thematic works. The countries of Africa and Asia had less than 100 publications, except for Japan and Taiwan, which published 226 and 128 items, respectively.

When analyzing the chronological development of the global research, the year 1962 can be identified as a starting point of activity with a slight rising of the scientific productivity concerning bacterial meningitis afterwards (Figure 1B). In 1990, this tendency has reached the number of 99 published items, whereas in the subsequent year, the numbers experienced an enormous rise of 150% with 248 publications. Except for the years of 1994, 1997 and 1998, which showed a slight decrease of 15%, the trend of the rising number of scientific items on bacterial meningitis kept going on until 2006, when 530 articles were published. This was followed by a decrease to 490 items in 2007.

#### 3.1.2. Development of Citations

The analysis shows that the global citation development stagnated at a low level until 1965 with a maximum of 60 citations per year. The years 1953, 1959 and 1962 were minor exceptions. The time interval between 1965 and 1991 was characterized by a strongly fluctuating development. However, a rising tendency can be noted: 994 received quotations can be dated to 1965; this was the first time the number of citations rose above 200. With the prior year counting 162 citations, the rise equals 514%. This, for that time, high level remained in the following two years, which had quotation numbers below 60. The years of 1968 and 1969 showed an increase in citations to 153, respectively 303. This pattern of increasing and decreasing numbers with an overall slight rise of citations was repeated until 1991. 1981 was the first year to reach the level of 1965 again, with 1040 quotations. The years 1991 and 1992 showed an enormous increase of 400%, with 9,825 citations in 1992 being the highest number of this investigation. Over the next six years, there were strong fluctuations of the development again, with significant increases and decreases discernible. A clear decrease of 32% in the development can be noted in 2001.

**Figure 2:**
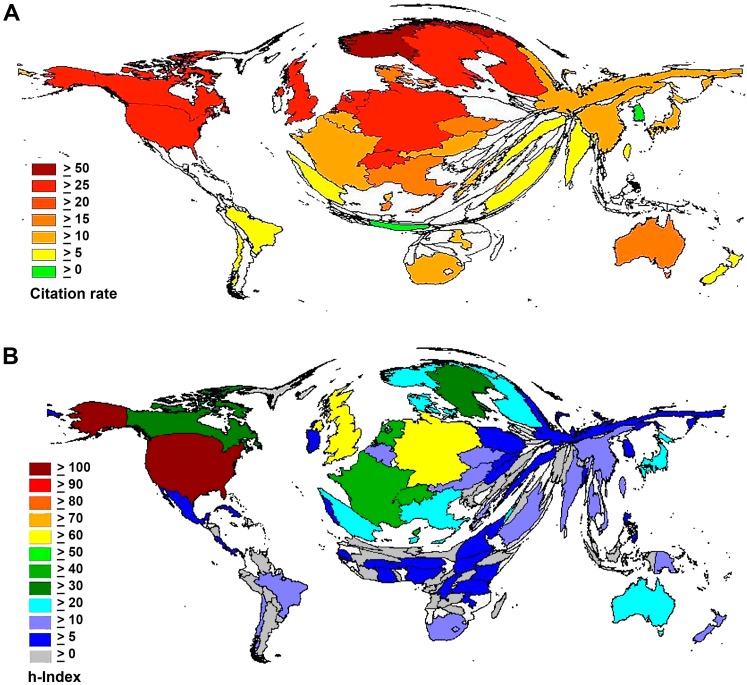
Citation parameters. (**A**) Density-equalizing map illustrating average citation rate. Colors encode average citation rates. (**B**) Density-equalizing map illustrating international h-indices (Hirsch-factor). Colors encode h-index values.

The analysis of the international citation rates lead to a ranking with Norway at the top. It had an average citation rate of 26.36 (Figure 2A). It was followed by Finland, which showed a citation rate of 24.16. The U.S. was able to reach an average of 24.06, while the European countries, Switzerland, The Netherlands, Sweden and the UK, possessed rates of 23. The citation rate of Germany was 21.75 and the rate of Canada 20.46. The rates of France and Japan (11.68 and 11.54, respectively) were on a similar level as Russia and China, with rates of 11.92 and 10.10.

#### 3.1.3. Comparison of Modified International h-Indices

The analysis of the country-specific h-index within the total number of identified articles shows the dominance of the U.S. (Figure 2B). Its h-index equals 106 and was the only one above 100. The UK followed with an h-index of 69, while Germany possessed a value of 61. The analysis shows that the h-indices of countries from Africa, Asia and East-Europe were less than 20 and, therefore, appeared compressed in the illustration (Figure 2B).

#### 3.1.4. Development of Cooperation

**Figure 3:**
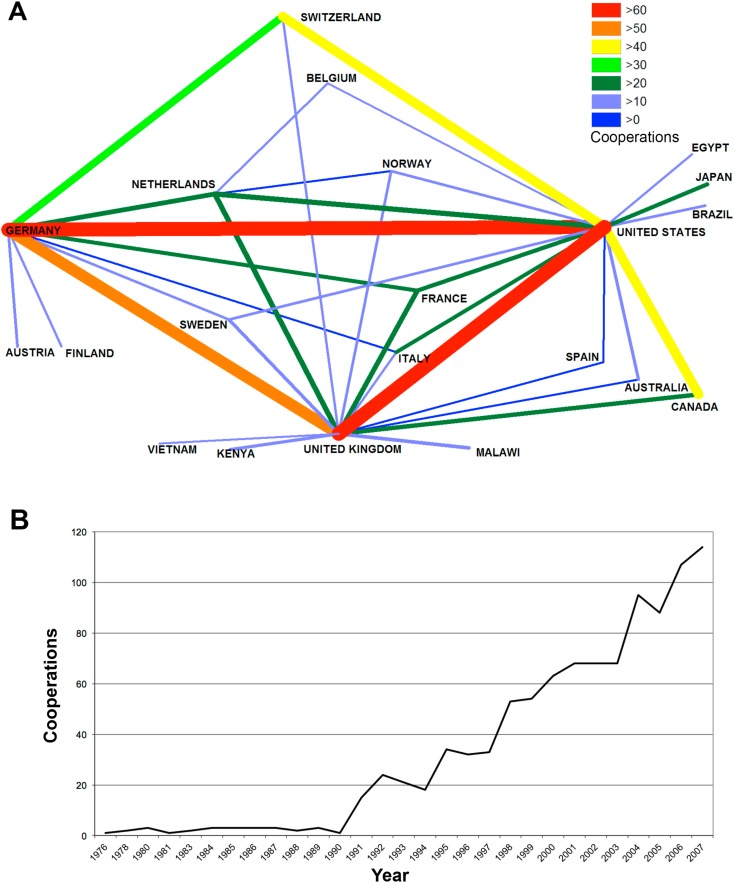
Cooperation. (**A**) Spider chart of international cooperation. Line thickness and color indicate the amount of cooperation between countries. Colors and line thickness indicate the numbers of bilateral cooperation. (**B**) Development of cooperation over a time period of 31 years.

The development of cooperation was measured over a time period of 31 years, beginning from 1976 to 2007 (Figure 3). There was a dichotomy present: Before 1991 the numbers of collaborations were stagnating, and after, a steady increase has been registered. The maximum number of articles that are based on international cooperation can be said to be three before 1991. There were minor fluctuations; however, the overall level of articles was stable for 15 years. Eventually, there was a strong rise of 400% of the amount of articles in 1991; 15 articles can be dated to that year. The productivity of international cooperation was rising further, after a drop of 25% in the years of 1993 and 1994. In the following years, a rising tendency can be noted, which results in 63 published items from the year 2000 published in an international cooperation. The ascending trend continued until 2007 in which, with 114, the highest number of this measurement has been achieved. The only exceptions to this development were the years 2001 to 2003, in which the amount of items was stable, and 2005, in which the article count dropped by 7%. Overall, the published items that result from international cooperation has been centupled over the last 31 years.

#### 3.1.5. Analysis of International Cooperation

The U.S. was the strongest cooperation partner in the network of scientific collaborations. In the observed period of time, the U.S. has published 70 items dealing with bacterial meningitis in cooperation with both Germany and the UK (Figure 3A). There was also a strong cooperation between Germany and UK present that resulted in 59 common articles. In the third place was cooperation between the U.S., Canada and Switzerland, with 49 cooperative efforts each. In total, the U.S. has cooperated with the largest amount of different countries (15), followed by the UK (14), whereas Germany has cooperated with nine different countries.

In principle, the trend of international cooperation works is obviously increasing over time (Figure 3B).

### 3.2. Discussion

Bacterial meningitis is still a major threat for public health settings. Despite the enormous scientific advances that have been made in recent years, there are still numerous outstanding questions to be targeted by research activities. Despite these requirements, there is no information available on the global research trends regarding this thematic issue. Therefore, the methods provided by the NewQIS platform [17,18] were used to carry out a combined density-equalizing mapping and scientometric analysis.

Therefore, this aims also to illustrate a sketch of the global meningitis research architecture using a large database, such as WoS. However, this analysis cannot be regarded as completely representative of the global meningitis research activity, since no database includes the entirety of all publications related to meningitis. Nevertheless, the WoS database is among the largest global biomedical databases, so that it can be hypothesized that the present findings should indicate the common global trends of meningitis research.

The results show a chronological increase in scientific productivity regarding the total number of publication. While this overall process can be described as steady over a time period of 60 years, it was boosted at the beginning of the 1990s, which seems to prevail into the present time, reflecting the higher standards of research, the advances in the understanding of the disease and the higher public interest in it. Additionally, the observed increase is due to the inclusion of abstracts and keywords to the database search strategies. This enlarged the pool of publications that could be retrieved on the issue of bacterial meningitis.

However, the amount of scientific works that were retrieved from the WoS database is only specific for a definite date. More recent studies should reveal a different number of entries, because, for one thing, the number of listed journals is increasing over time, for another thing, the new WoS database, Core Collection, which was introduced with the new interface of the Web of Science at the beginning of 2014, allows searching in more indices,* i.e.*, the Book Citation Index or the Conference Proceeding Citation Index.

Second, a language bias is present within WoS and other biomedical databases, such as PubMed or Scopus. In this respect, journals published in English have a higher chance of getting included in the databases [27], and therefore, non-English speaking countries may be underrepresented concerning their research activities. However, it should be noticed that at the present time, high quality studies are usually published in journals with English as the language. Albeit, important, but regional, data, such as regional epidemiologic information about meningitis, are probable often published in non-listed journals. Moreover, it should be noticed that traditional journals are included more frequently than novel journals, which may have the same quality standards [28]. These aspects indicate the necessity of a precise discussion of methodological issues.

Third, the quality indicators that we applied,* i.e.*, total citations, citation rate, country-specific h-indices, need to be discussed critically. In this respect, there are numerous problematic issues present with these kinds of indicators. Therefore, the data should not be over interpreted concerning research quality, as discussed earlier [26,27,28]. Assessing the actual quality of a research study is only possible by advanced meta-analysis using,* i.e.*, Cochrane approaches [29].

Fourth, the Matthew effect needs to be taken into account, since communication systems in science are directed towards a reward of highly productive and renowned scientists and institutions. This leads to a pyramidal citation scheme [30].

The results of this study show that most publications on the subject of bacterial meningitis derive from the U.S., followed by the UK, Germany and France. This dominance can also be found in international cooperation, which is being led by Germany, the U.S. and the UK. The increasing growth in articles concerning bacterial meningitis at the beginning of the early 1990s is accompanied by an accretive development of international cooperation.

Similar patterns of research activity are present in numerous other fields of biomedical science, including areas such as internal medicine [31,32,33,34] and various aspects of public health with tobacco issues [20,35] or even drowning accidents [36].

Interestingly, a variety of other infectious diseases have been analyzed using similar approaches.

In hepatitis research,* i.e.*, a similar global activity pattern was present [37]: in the time span of 1971 to 2011, more than 49,000 articles were published by 250 countries, of which the U.S. was the most productive supplier, with 28% of all publications. In this analysis, the co-leading countries, Germany, the UK, Japan and France, were added to by China.

In a separate study addressing global influenza research [38], the U.S. dominated also the global research in a set of 51,418 identified influenza-related publications. They participated in more than 37% of all publications, followed by the UK and Germany, with more than 5% [38].

In striking contrast to these “westernized” research patterns are the studies on infectious diseases that are linked to neglected tropical diseases, including yellow fever [39] or leishmaniasis [40]. The research pattern of these diseases includes a country placed in the top two, which is not present in the other density equalizing mapping analyses: in a total of 5053 yellow fever-associated publications published by 79 countries, the U.S. (751) was followed by Brazil (203) [39]. A similar picture was also present with leishmaniasis: here, the phalanx of the most productive countries included Brazil at the second and India at the fifth position, with 2049, respectively 1011, publications, while the U.S., as the most active country, had 4905 publications, in a total set of 19,288 identified articles [40].

When focusing on the qualitative parameters, the citation analyses reveal that the highest activity of research is not concomitant with the highest citation rates. In this respect, the nation with the highest average citation rate is Norway, followed by Finland, the U.S. and Switzerland. However, the U.S. holds the most total citations and the highest h-index, followed by Germany and the UK.

## 4. Conclusions 

The present study is the first thorough analysis of bacterial meningitis research and gives precise information about the global research architecture. Since the funding of research depends on scientometric parameters, the importance of analyses that are directed on this issue cannot be denied. The assessment of the global scientific output is getting more and more confusing, due to the enormous increase of journals and publications. In this respect, the application and the advancement of the scientometric tools and parameters are imperative. All of the analyses within the NewQIS platform should be seen in this context.

As seen in scientometric analysis on other diseases, the U.S. and other highly developed countries are leading concerning individual research activity. However, countries with high case numbers, but lower development levels, also need to focus on this area of biomedicine in the future, potentially with customized public health-related research programs, since therapeutic and diagnostic advances are extremely expensive and already explored by the U.S., Europe and some highly developed Asian countries, such as Japan. Thus, further investigations are needed to assess the future development in this respect.
